# Investigating dose-dependent effects of chemical compounds targeting rumen fermentation pathways using an in-vitro rumen fermentation system

**DOI:** 10.1186/s12866-025-03969-7

**Published:** 2025-05-27

**Authors:** Rajan Dhakal, André Luis Alves Neves, Rumakanta Sapkota, Prabhat Khanal, Lea Ellegaard-Jensen, Anne Winding, Hanne Helene Hansen

**Affiliations:** 1 Department of Veterinary and Animal Sciences; Section for Animal Health and Welfare, University of Copenhagen, Frederiksberg C, DK-1870 Denmark; 2 Department of Environmental Science, Faculty of Technical Sciences, Aarhus University, Roskilde, DK-4000 Denmark; 3 Faculty of Biosciences and Aquaculture, Nord University, Skolegata 22, Steinkjer, 7713, P 340 Norway

**Keywords:** Fermentation kinetics, Amplicon sequencing, Euryarcheota, Firmicutes, Methanogenesis, Reductive acetogenesis, Propionigenesis

## Abstract

**Background:**

Ruminal fermentation leads to the formation of methane (CH_4_) as a byproduct, which is one of the major greenhouse gases. Despite extensive research efforts involving the use of various anti-methanogenic and hydrogen sink compounds, the current understanding of the dose-response effects of these compounds on the rumen microbiome and fermentation profile is limited. In this study, potential methanogenesis inhibitors or electron acceptors were evaluated for their effects on methane production, fermentation, and prokaryotic community composition. Dose-response effects of sodium 2-bromoethanesulfonate (BES: 0, 2.5, 5, 10 mmol/L), p-hydrocinnamic acid (HoC: 0, 5, 10 mmol/L), and sodium fumarate dibasic (DFS: 0, 5, 10, 20 mmol/L) on dry matter degradation, total gas production, methane concentration and yield, composition and yield of volatile fatty acids, and prokaryote composition were studied during 48 h rumen fermentations.

**Results:**

The BES decreased the yield (ml/ g DM) and concentration (%) of CH_4_, acetic, isobutyric, and total VFA (t-VFA) concentrations (mmol/g DM), and increased propionic and butyric acid concentrations (mmol/g DM) without affecting dry matter degradability (dDM) as the dose increased. The HoC decreased dDM, total gas production (TGP), CH_4_ yield (ml/ g DM) and increased tVFA concentration (mmol/g DM) as the dose increased. The increasing dose of DFS increased the pH, propionic acid and tVFA concentrations (mmol/g DM) and decreased the yield (ml/ g DM) and concentration (%) of CH_4_ without affecting dDM. Sodium 2-bromoethanesulfonate, HoC, and DFS doses did not significantly change the alpha-diversity and beta-diversity indices of the prokaryotic communities at the amplicon sequence variant level, although the relative abundances of specific phyla were affected by the treatments. The major bacterial phyla across all samples were Bacteroidetes, Proteobacteria, Firmicutes, Spirochaetota, Verrucomicrobiota, and Patescibacteria.

**Conclusions:**

This study demonstrated that (i) all the evaluated compounds affected the targeted metabolic pathways without influencing the structure of the rumen microbial community, (ii) BES inhibited methanogenesis without affecting dry matter degradability, and (iii) HoC and DFS shifted hydrogen utilization towards acetate and propionate production. The recommended doses, to reduce methane during in-vitro rumen fermentation for BES, HoC, and DFS were determined to be 2.5 mmol/L, 5 mmol/L, and 10 mmol/L, respectively. Further research is suggested to understand the interactive effects of methane inhibition compounds, such as BES, in conjunction with H_2_ sink compounds such as HoC and DFS. However, caution is advised when using halogenated compounds like BES, as some methanogens have developed resistance and BES is not approved for use as a feed additive for live animals.

**Supplementary Information:**

The online version contains supplementary material available at 10.1186/s12866-025-03969-7.

## Introduction

Ruminants are an integral part of human civilization as they have been providing different services and products for humans for centuries at the expense of consuming low-quality fibrous plant material with no nutritious value for humans. The digestive system of ruminants is highly specialized and complex, with the rumen being the main site of feedstuff fermentation. In ruminant animals, microbial fermentation in the rumen breaks down the ingested fiber sources into metabolites and nutrients that are beneficial to the host animal [1]. In the fermentative process, rumen microbes produce volatile fatty acids (VFAs), mainly acetate, propionate, and butyrate, and gases such as CO_2_, H_2_ and CH_4_. The accumulation of H_2_ can thermodynamically and kinetically inhibit fermentation in the rumen [2]. Therefore, H_2_ needs to be disposed of and kept at low concentrations in the rumen to promote fermentation. The VFA and microbial biomass produced during rumen fermentation contribute more than ~ 70% of the required energy and ~ 60% of the needed protein to the host [3]. However, the CH_4_ produced is released into the atmosphere through eructation, contributing to a significant portion of global methane emissions [4]. Methane is estimated to have approximately 28 times the global warming potential of CO_2_ over a 100-year period, raising doubts regarding the sustainability of ruminant production [5, 6]. The amount of CH_4_ produced during the rumen fermentation depends on different factors, such as the animal type, quantity, feed chemical composition, and quality, additives in the feed, and pre-probiotics used to feed the animals. The production of CH_4_ during rumen fermentation depends upon the H_2_ partial pressure in the rumen, which provides the ideal environment for the archaeal microbes to thrive [7].

During rumen fermentation, there are three primary pathways for the accumulated H_2_ disposal: (a) methanogenesis, (b) reductive acetogenesis, and (c) propionigenesis [8]. Methanogenesis is the major pathway for H_2_ sinks in the rumen, but it results in a loss of 2–12% energy [9]. One of the strategies for mitigating CH_4_ emissions is to inhibit methanogenesis, but it may lead to the accumulation of dissolved H_2_ in the rumen.

Methanogens are highly efficient at utilizing H_2_ in typical rumen environments compared to other microbes [7]. One effective strategy to enhance the efficiency of competing microbes for H_2_ is to inhibit methanogens or provide alternative electron acceptors for reductive acetogenesis and propionigenesis [10]. This approach is more desirable than solely inhibiting methanogens, as it can potentially shift H_2_ utilization towards the production of beneficial metabolites like acetate and propionate [11]. Some electron acceptors, such as nitrate, sulfate, p-hydrocinnamic acid, and di-sodium fumarate, have been observed to thermodynamically outcompete methanogens [8, 12–15]. However, fumarate can also be metabolized into acetate via the malate-pyruvate pathway in the rumen [14]. These electron acceptors are either present in very low concentrations or unavailable in the feedstuff. A possible alternative is to provide these compounds as feed additives.

Despite extensive research efforts involving the use of various anti-methanogenic and hydrogen sink compounds, the current understanding of the dose-response effects of the chemical compounds investigated in this study on the rumen prokaryote and fermentation profile is limited. The present study aimed to explore the effects of diverse doses of compounds targeting the pathways of CH_4_, acetate, and propionate-producing microbes in the rumen. Sodium 2-bromoethanesulfonate (BES) was used to target methanogenesis, whereas p-hydrocinnamic acid (HoC) and sodium fumarate dibasic (DFS) were used as electron acceptors to target reductive acetogenesis and succinate/randomizing (propionigenesis) pathways. We hypothesized that the CH_4_, acetate, and propionate-targeting compounds significantly affect the microbial composition, fermentation kinetics, and fermentation parameters in in-vitro rumen fermentation. The objectives of this study were (1) to assess the dose-response of the compounds BES, HoC and DFS on dry matter degradability, total gas production, concentration and yield of CH_4_, composition of volatile fatty acids and prokaryotic microbial composition; and (2) to find the most effective dose for the compounds targeting methane, acetate, and propionate pathways.

## Materials and methods

### Treatments

Sodium 2-bromoethanesulfonate (BES; CAS: 4263-52-9) and sodium fumarate dibasic (DFS; CAS: 17013-01-3) were tested in four different doses (BES: 0, 2.5, 5, 10 mmol/L; DFS: 0, 5, 10, 20 mmol/L ), and p-hydrocinnamic acid (HoC; CAS: 501-98-4) was tested in three different doses (0, 5, 10 mmol/L) in three different runs, with each dose in each run tested in triplicate (Supplementary Table S1) during fermentations using maize silage (MS) as a substrate (0.5 g, DM%: 93.05, NDF%: 44.81%, ADF%:24.86, ADL%:1.86, and CP: 8.3%) in 90 ml of rumen inoculum (rumen fluid and buffer). The amount of additive in each treatment was chosen to reflect the maximum and least amount of additive that could be used from the previous studies [8, 12–16]. Previously Agarwal et al. [16] used 5mMol/L BES, Cord-Ruwish et al. [12] used 5 mMol/l HoC and Newbold et al. [13] used 640 g ~ 10 mMol/L of DFS in in-vitro fermentation or pure culture to inhibit the methanogens. The BES and DFS were purchased from Sigma-Aldrich, and HoC was purchased from Acros-Organics. The HoC and DFS was pre mixed with ethanol before adding to the bottle in order to increase the solubility of the compound and same about of the ethanol was also added to the maize silage (MS) of these experiment.

### In-vitro fermentation

The use of two rumen-fistulated Jersey heifers was authorized by the Danish Animal Inspectorate (license nr. 2012-15-2934- 00648). The heifers were fed ad libitum haylage (85% DM, 7.5 MJ/kg metabolizable energy, and 11% CP) for over six weeks before the experiment. For each fermentation, animals fasted for 12 h before sampling, and water was removed 2 h before rumen fluid sampling [17]. Rumen fluid, including particulate matter, was collected from the same cannulated heifers in all experiments at the Large Animal Hospital of the University of Copenhagen (Taastrup, Denmark).

A four-part buffer solution was prepared as described by Menke et al. [18] and further in-vitro fermentation was carried out as described by Vargas-Bello-Pérez et al. [19]. The collected rumen fluid was filtered and gently squeezed to collect microbes attached to the feed particles through two cheesecloth layers and added to the buffer in a 1:2 ratio. Each bottle was fitted with an individual module (ANKOM^RF^ Technology, Macedon, NY, USA), which sends pressure measurements via a receiving base station to an attached computer. The software was programmed to release gas from the headspace in the bottles at 0.75 psi through a vent valve. The gas produced during the fermentation was collected in a gastight (SKC, Flex Foil PLUS) bag attached to the vent valve tube of the module. The modules were incubated in a thermoshaker (Gerhardt, Königswinter, Germany) at 39 °C at 40 rpm. Bottles with rumen fluid but without feed (blank) were included to determine baseline fermentation as described by Menke et al. [18]. The experiment was stopped after 48 h by placing the bottles on ice, and the pH of the rumen fluid of each bottle was measured (HECH pH31^®^) before collecting the undigested residue in a pre-weighed filter bag with a porosity of 25 μm (Ankom F57) using a vacuum suction pump (maximum of 10 psi) (Supplementary Figure S8). To examine the microbial composition and quantify volatile fatty acids (VFAs), 10 ml of filtered rumen fluid was collected in a 12 ml Falcon (Sarstedt) tube and stored at -20 ^o^C until further analysis. After filtration of the fermented feed, the filter bags were first air-dried at room temperature for 24 h, then dried at 100 °C for 2 h, according to the ANKOM protocol [20], cooled to ambient temperature in a desiccator, and weighed.

### VFA and methane determinations

The thawed rumen fluid at room temperature was mixed with a metaphosphoric solution (5:1 ratio), and crotonic acid was added as an internal standard. The well-suspended mixture was incubated for 30 min at room temperature and centrifuged at 14,000 rpm for 10 min. Then, the supernatant was filtered through a syringe filter with a 0.2 μm pore diameter (MiniSart Syringe Filter, Satorius), and a 1 ml filtered sample was collected in 2 ml gas chromatography (GC) vials and analyzed. The VFA composition was determined by GC-FID (Nexis GC-2030, Shimadzu Scientific Instruments Inc., Kyoto, Japan) as described by Dhakal et al. [21]. The methane concentration in the gas-tight bags was measured as described by Dhakal et al. [22] directly after a 48-hour incubation in a gas chromatograph (GC-TDC) (Agilent 7820 A GC, Agilent Technologies, Santa Clara, CA, USA). The total methane volume (% of collected gas) produced was then calculated.

### Microbiome analysis

Two ml of the thawed rumen fluid was transferred into a sterile tube and centrifuged at 15,000 g for 10 min to obtain pellets for genomic DNA extraction. DNA from the pellets was extracted using Bead-Beat Micro Ax Gravity (A&A Biotechnology, Gdynia, Netherlands). The concentration and purity of the extracted DNA were measured with a NanoDrop Lite UV-Vis spectrophotometer (Thermo Fisher Scientific, Waltham, MA, USA).

The prokaryote primers 515 F (GTGCCAGCMGCCGCGGTAA) and 806R (GGACTACHVGGGTWTCTAAT) [23] with Illumina Nextera overhang adapters were used to amplify the V4 region of the prokaryote 16S rRNA gene region. The first and second PCR runs were performed as described earlier [24]. After the second PCR product was obtained, gel electrophoresis (1.5% agarose) was performed with each sample to ensure successful amplification. Then, the amplicons were cleaned using HighPrep magnetic beads (MagBio Genomics Inc. Gaithersburg, USA) according to the manufacturer’s instructions. Finally, amplicons were pooled in equimolar concentrations, and sequencing was performed using the Illumina MiSeq platform at Dep. of Environmental Science, Aarhus University.

### Calculations and statistical analysis

Gas production and dry matter residues from the blank treatments were used to correct the gas production, methane concentration, and dry matter degradation. Subsequently, the results from the blanks were discarded, and only the results of the blank-corrected variables were used in the calculations. As described by Dhakal et al. [17], the ideal gas law was used to convert the cumulative pressure to ml gas at standard temperature and pressure (STP), and volume of gas produced per gram of dry matter and dry matter disappeared (dDM, mg/100 mg) were calculated.

Statistical analyses were performed using R v. 4.0.3 (https://www.R-project.org/), using the ‘*emmeans*’ package for orthogonal contrasts to test the linear and quadratic effects of dose [25]. Comparison of the means of the dose response with those of the control (maize silage; MS) was performed for each of the three compounds as per the statistical model below

$$Yijk\, = \,\mu \, + \,Ti + \,Rj\, + \,Eijk$$

Where *Y*_*ijk*_ corresponds to the *ijk* observation, *Ti* corresponds to treatments (i = concentrations of the chemicals), *µ* is the general mean, *R*_*j*_ is the random effect (batch of fermentation), and *E*_*ijk*_ corresponds to the ijk observation related error.

### Bioinformatics

The DNA reads obtained from the Illumina MiSeq run were analyzed using QIIME2 [26] and the DADA2 plugin for quality control [27]. In brief, the paired-end reads were denoised, joined, dereplicated, with forward and reverse primer trimmed, and chimeras were filtered using the ‘dada2 denoise paired’ command. Following this, taxonomy to amplicon sequence variants (ASV) were assigned using ‘feature-classifier classify-consensus-vsearch’ using the SILVA 138 database [28]. The ASV table and taxonomy files was imported into R version 4.0.3 [29] to perform data analysis and visualization. To address disparities in library sizes between samples, an alternative normalization procedure was implemented using the “rarefy_even_depth” command of the R package ‘phyloseq’ to rarefy the data. Diversity-based analysis was performed using ‘vegan’ v. 2.5-7 [30] and ‘phyloseq’ version 1.34 [31]. Alpha diversity was measured using observed richness and the Shannon diversity index, while beta diversity was estimated with Bray-Curtis distance matrices. Bray-Curtis distances were visualized using principal coordinate analysis (PCoA). The variance partitioning and significances of experimental factors were performed by permutation analysis of variance (PERMANOVA). Further, pairwise Adonis tests were performed to evaluate the differences against each pair of treatment. The ‘DESeq2’v. 1.40.2 package in R was used to detect the prokaryotic species that displayed the most significant changes in differential abundance across treatments using pairwise comparison.

## Results

### 16S rRNA gene amplicon sequencing

After quality control and removal of chloroplast and mitochondria reads of the Illumina MiSeq amplicon sequencing, a total of 1,038,930 reads were obtained, which comprised 3,930 ASVs. Following filtration, each sample had an average of 23,087 ± 8,081 reads.

### Rumen fermentation characteristics and prokaryote composition and structure of sodium 2-bromoethanesulfonate (BES)

We found that increasing doses of BES influenced fermentation parameters (Table 1). Increasing doses of BES showed both a linear (*p* < 0.05) and quadratic (*p* < 0.05) relationship with yield and concentration (%) of methane, concentration of acetic, propionic, isobutyric, and butyric acids, and total VFA (tVFA), while pH and valeric acid showed a significant (*p* < 0.05) linear decrease.

**Table 1:** In-vitro fermentation characteristics of maize silage and VFA profiles of increasing doses of sodium 2-bromoethanesulfonate (BES)

Dose (mmol/L)	0	2.5	5	10	SEM	Linear	Quadratic
pH	6.84	6.75	6.77	6.77	0.015	0.0034	0.0038
TGP ml/g DM*	211	174	185	186	9.72	0.6194	0.04
dDM %*	80	80	81	81	0.85	0.2753	0.82
CH_4_% of TGP*	9.0	0.02	0.01	0.0	0.38	< 0.0001	0.0001
CH_4_ ml/g DM*	18	0	0.212	0.071	1.42	0.0038	0.02
VFA (mmol/L)							
Acetic*	12.60	9.08	8.66	8.76	0.37	< 0.0001	< 0.0001
Propionic*	5.72	7.35	7.04	6.89	0.26	< 0.0001	< 0.0001
Isobutyric*	0.15	0.10	0.1	0.09	0.008	< 0.0001	0.0001
Butyric*	1.90	2.30	2.30	2.20	0.12	0.0016	< 0.0001
Isovaleric*	0.25	0.18	0.22	0.19	0.03	0.0717	0.20
Valeric*	0.30	0.26	0.24	0.22	0.01	< 0.0001	0.33
tVFA*	20.90	19.20	18.60	18.40	0.64	< 0.0001	0.0069

DM: dry matter, VFA: volatile fatty acid, TGP: total gas production, tVFA: total VFA, SEM: largest standard error of the mean, dDM: disappeared dry matter. *The values present in the table are blank corrected

**Fig. 1:**
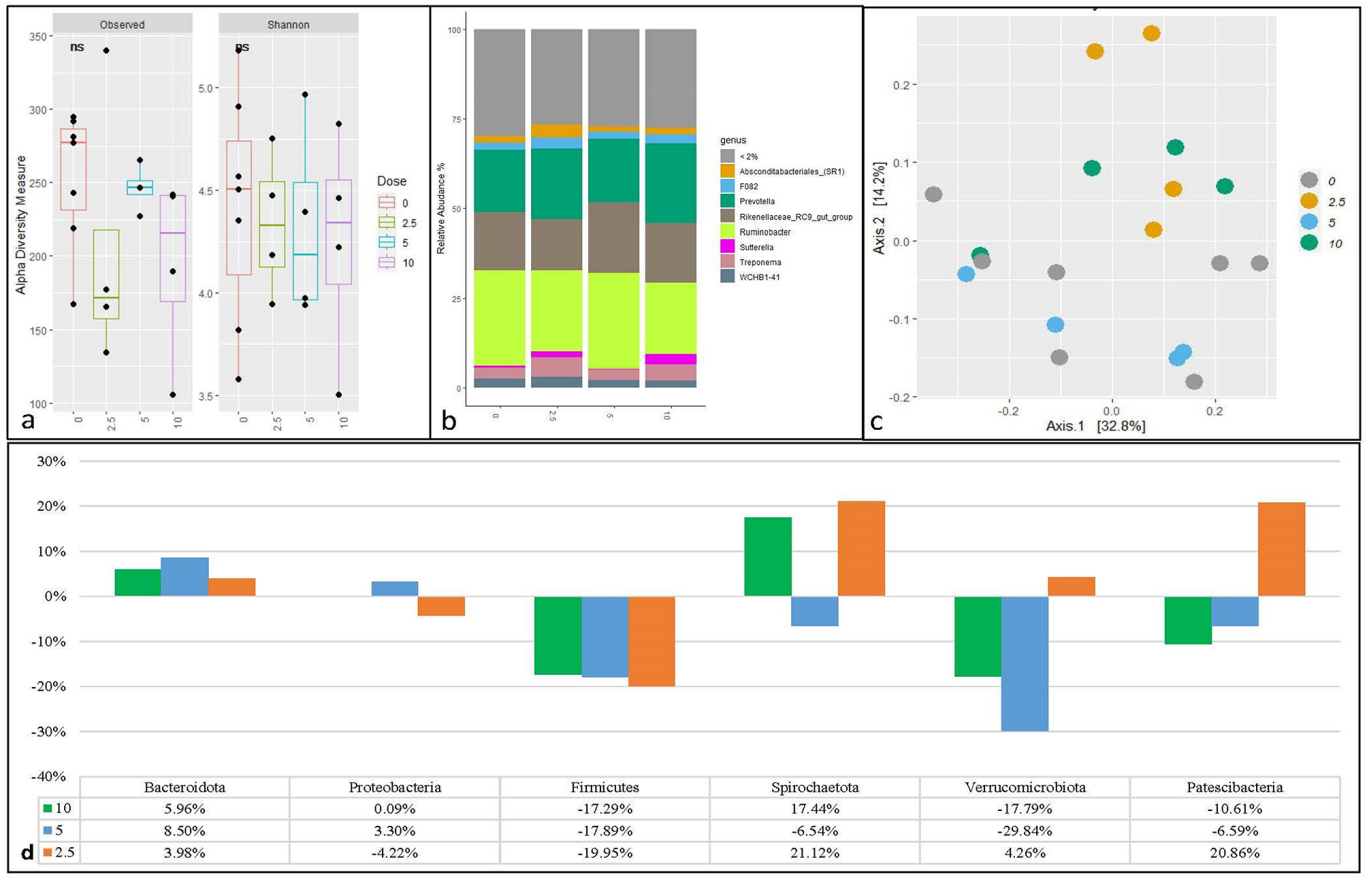
Prokaryote diversity and abundance in in-vitro fermentation of maize silage with four doses of sodium 2-bromoethanesulfonate (BES). Alpha diversity measured by observed richness and Shannon index (**a**), relative abundance of most abundant genus (**b**), beta diversity using Bray-Curtis distance dissimilarity matrices (**c**) and relative change in abundance of bacterial phyla from maize silage control (**d**)

Increasing doses of BES did not affect alpha diversity when measured by the Shannon index and species richness (observed) (Fig. 1a). The dominant phyla with an average > 1% relative abundance across all samples were Bacteroidota (47.5%), Proteobacteria (25.95%), Firmicutes (12.29%), Spirochaetota (5.88%), Verrucomicrobiota (3.66%) and Patescibacteria (3.06%) (Supplementary Figure S4). The genera with an average relative abundance >2% across all samples were *Ruminobacter*,* Prevotella*,* Rikenellaceae_RC9_gut_grup*,* Treponema*,* WCHB1-41*,* Sutterella*,* F082* and *Absconditabacteriales_(SR1)* (Fig. 1b). In the BES treatment, *Rikenellaceae_RC9_gut_group*,* Ruminobacter*,* UCG-002*,* F082*,* SP3-e08*,* WCHB1-41*,* Treponema*,* and probable_genus_10* were the major enriched genera (Supplementary Table S2). A PCoA plot of Bray-Curtis distance dissimilarity matrices showed clustering of prokaryotic community structure based on 2.5 and 5 doses of BES, however no clear pattern was seen for control and dose 10 (Fig. 1c). The overall effect of BES dose, investigated by the PERMANOVA test, was non-significant (*p* > 0.05). However, a comparison of only the 2.5 and 5 doses, by the pairwise Adonis test, showed a significant difference (*p* < 0.05). While evaluating community at phyla level, the abundances of Planctomycetota at the phylum level were significantly different (*p* < 0.05) between different doses (Supplementary Fig. 1). Compared to the control group (dose 0) (Fig. 1d), BES administered at different doses had varying effects on the relative abundance of different phyla. Specifically, the relative abundance of Bacteroidota increased, while the Firmicutes relative abundance decreased. The effect on Proteobacteria, Spirochaetota, Verrucomicrobiota, and Patescibacteria was inconsistent when compared to the control group.

### Rumen fermentation characteristics and prokaryote composition and structure of p-hydrocinnamic acid (HoC)

Increasing doses of HoC showed both a linear (*p* < 0.05) and quadratic (*p* < 0.05) relationship with TGP, dDM, methane yield and tVFA (Table 2). On the other hand, pH, methane concentration, and concentrations of propionic, isobutyric, butyric, isovaleric, and valeric acid showed a significant linear decrease (*p* < 0.05), while a quadratic effect (*p* < 0.05) was observed with acetic acid.

**Table 2:** In-vitro fermentation characteristics of maize silage and VFA profiles of increasing doses of p-hydrocinnamic acid (HoC)

Dose (mmol/L)	0	5	10	SEM	Linear	Quadratic
pH	6.83	6.75	6.76	0.03	0.0172	0.086
TGP ml/g DM*	261	286	242	15.7	0.002	< 0.0001
dDM %*	77.7	74.9	66.6	1.59	0.0007	0.013
CH_4_% of TGP*	13.2	12.3	10.2	1.16	< 0.0001	0.13
CH_4_ ml/g DM*	32.9	37.2	26.2	4.8	0.044	0.0069
VFA (mmol/L)						
Acetic*	19.4	22.5	20.1	1.26	0.1115	< 0.0001
Propionic*	5.84	4.98	4.31	0.12	< 0.0001	0.1682
Isobutyric*	0.078	0.063	0.033	0.01	< 0.0001	0.23
Butyric*	1.89	1.74	1.58	0.23	0.0005	0.99
Isovaleric*	0.093	0.053	0.026	0.02	< 0.0001	0.45
Valeric*	0.179	0.153	0.128	0.0129	< 0.0001	0.91
tVFA*	27.5	29.5	26.2	1.34	0.0033	< 0.0001

DM: dry matter, VFA: volatile fatty acid, TGP: total gas production, tVFA: total VFA, SEM: largest standard error of the mean, dDM: disappeared dry matter. *The values present in the table are blank corrected

**Fig. 2:**
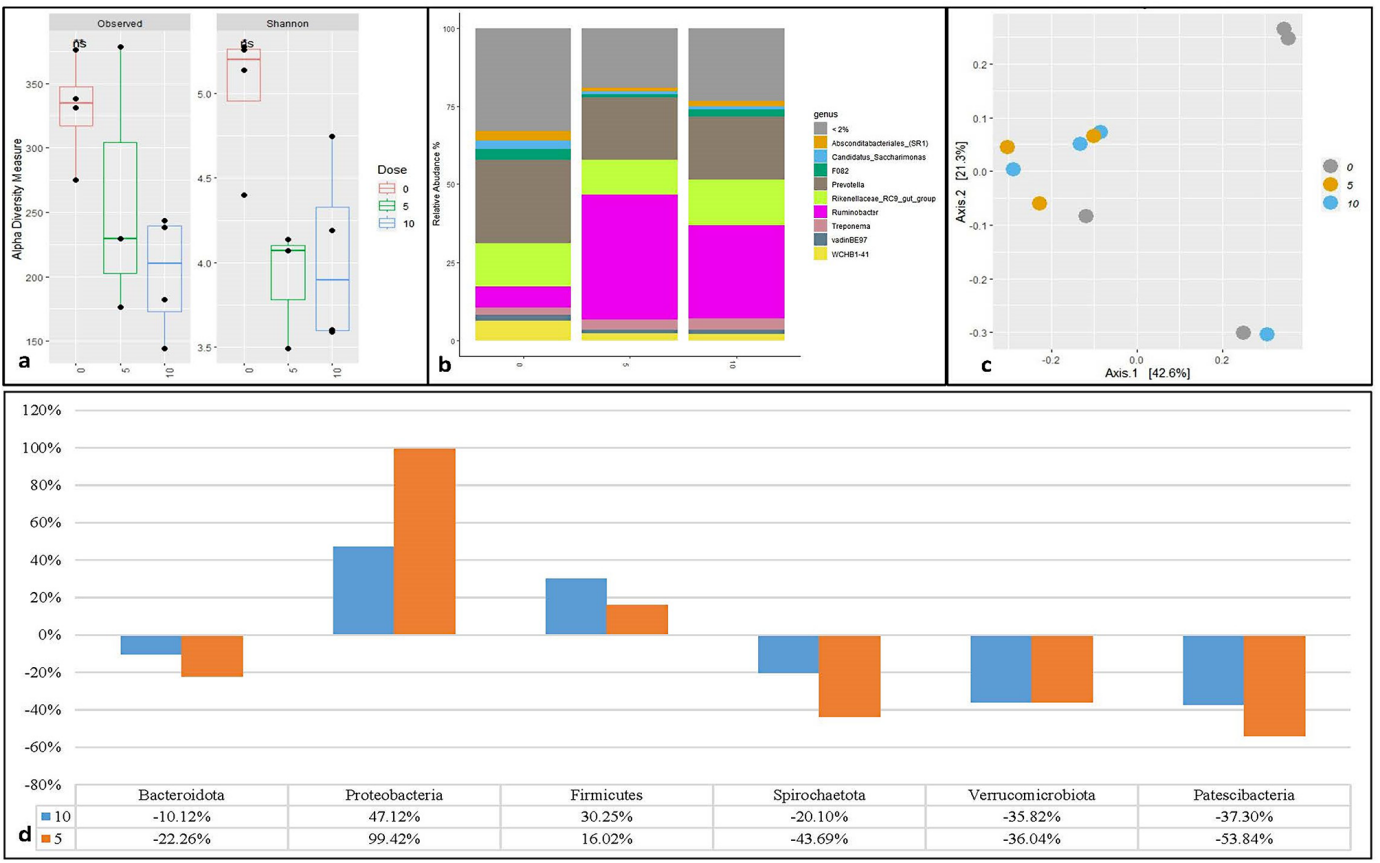
Prokaryote diversity and abundance in in-vitro fermentation of maize silage with three doses of p-hydrocinnamic acid (HoC). Alpha diversity measured by observed richness and Shannon index (**a**), relative abundance of most abundant genus (**b**), beta diversity using Bray-Curtis distance dissimilarity matrices (**c**) and relative change in abundance of bacterial phyla from maize silage control (**d**)

**Fig. 3:**
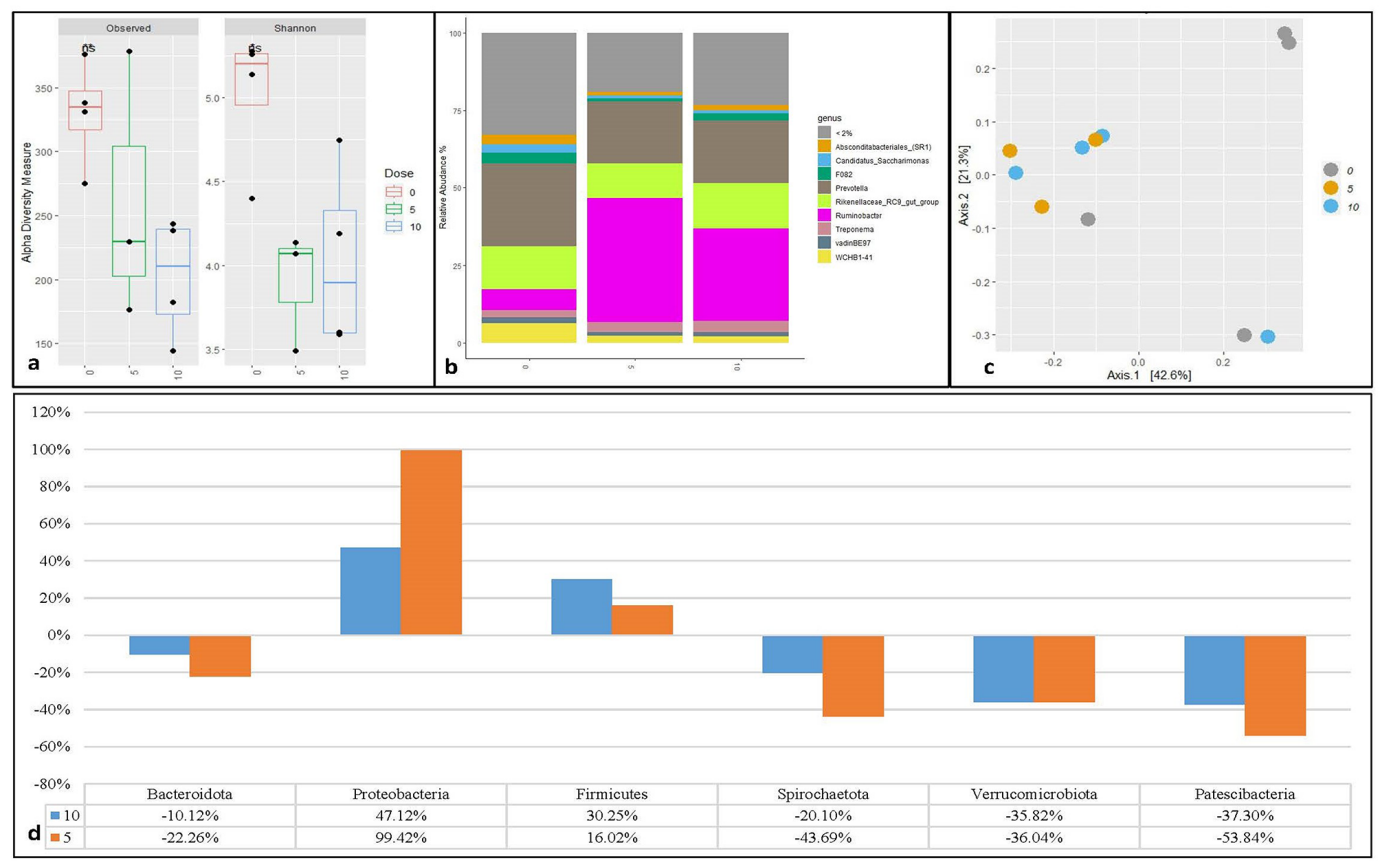
Prokaryote diversity and abundance in in-vitro fermentation of maize silage with four doses of sodium fumarate dibasic (DFS). Alpha diversity measured by observed richness and Shannon index (**a**), relative abundance of most abundant genus (**b**), beta diversity using Bray-Curtis distance dissimilarity matrices (**c**) and relative change in abundance of bacterial phyla from maize silage control (**d**)

Increasing doses of HoC did not affect alpha diversity (Fig. 2a). The phyla with an average of > 1% across all samples were Bacteroidota (46.17%), Proteobacteria (28.55%), Firmicutes (9.91%), Spirochaetota (6.28%), Verrucomicrobiota (4.33%) and Patescibacteria (3.05%) (Supplementary figure S5). The genera with an mean relative abundance > 2% across all samples were *Absconditabacteriales_(SR1)*,* Candidatus_Saccharimonas*,* F082*,* Prevotella*,* Rekenellaceae_RC-_gut_group*,* Ruminobacter*,* Treponema*,* VadinBE97*, and *WCHB1-41* (Fig. 2b). In HoC, *F082*,* Prevotella*,* Ruminococcus*,* Prevotellaceae_UCG_001* and *UCG-002* were enriched genera (Supplementary Table S2). A PCoA plot showed no clear clustering of prokaryotic community structure based on the HoC dose (Fig. 3c). Further, PERMANOVA test revealed no significant difference (*p* > 0.05) between the prokaryotic community structures at different doses. However, the abundances of Verrucomicrobiota at the phylum level were significantly different (*p* < 0.05) between different doses (supplementary Figure S5). In comparison to the control (dose 0) (Fig. 2d), HoC at different doses had a positive effect on the relative abundance of Proteobacteria, and Firmicutes, and a negative effect on Bacteroidota, Spirochaetota, Verrucomicrobiota, and Patescibacteria compared with the control.

### Rumen fermentation characteristics and prokaryote composition and structure of sodium fumarate dibasic (DFS)

Increasing doses of DFS showed both a linear (*p* < 0.05) and quadratic (*p* < 0.05) relationship with yield and concentration of methane and tVFA, while, pH and concentration of propionic acid, linearly increased (*p* < 0.05) and concentrations of isobutyric, butyric, isovaleric and valeric acids linearly (*p* < 0.05) decreased (Table 3). A quadratic effect of dose (*p* < 0.05) was observed with TGP and the concentration of acetic acid.

**Table 3:** In-vitro fermentation characteristics of maize silage and VFA profiles of increasing doses of sodium fumarate dibasic (DFS)

Dose (mmol/L)	0	5	10	20	SEM	Linear	Quadratic
pH	6.83	6.86	6.91	6.93	0.02	0.0009	0.66
TGP ml/g DM*	259	245	243	255	18.9	0.6194	0.037
dDM %*	78	78	78	77	0.96	0.2753	0.82
CH_4_% of TGP*	13.22	8.8	6.33	7.66	1.75	< 0.0001	0.0001
CH_4_ ml/g DM*	32.6	23.9	17.3	21.7	5.69	< 0.0001	0.0002
VFA (mmol/L)*							
Acetic*	19	17.6	17.3	19	1.35	0.8811	0.036
Propionic*	6.09	9.0	11.91	13.03	0.597	< 0.0001	0.089
Isobutyric*	0.086	0.078	0.060	0.054	0.011	0.0001	0.921
Butyric*	1.92	1.83	1.63	1.46	0.097	< 0.0001	0.375
Isovaleric*	0.097	0.079	0.069	0.070	0.013	0.01	0.213
Valeric*	0.183	0.189	0.17	0.151	0.014	0.0353	0.309
tVFA*	27.4	28.7	31.2	33.9	1.63	< 0.0001	0.514

DM: dry matter, VFA: volatile fatty acid, TGP: total gas production, tVFA: total VFA, SEM: largest standard error of the mean, dDM: disappeared dry matter. *The values present in the table are blank corrected

Increasing doses of DFS (Fig. 3) did not affect (Fig. 3a) alpha diversity measures at the ASV level when measured by the Shannon index and species richness (observed). The phyla with an average of > 1% across all samples were Bacteroidota (48.90%), Proteobacteria (23.08%), Firmicutes (11.75%), Spirochaetota (5.81%), Verrucomicrobiota (4.30%), Patescibacteria (3.14%), and Cyanobacteria (1.36%) (Supplementary figure S6). The genera with an average relative abundance >2% across all samples were *Absconditabacteriales_(SR1)*,* Candidatus_Saccharimonas*,* Clostridia_UCH-014*,* F082*,* Gastranaerophilales*,* Prevotella*,* Rikenellaceae_RC9_gut_grup*,* Ruminobacter*,* Treponema*,* VadinBE97* and *WCHB1-41* (Fig. 3b). In DFS, *F082*,* Succinivibrionaceae_UCG-002*,* Prevotella*,* Butyrivibrio*,* Sutteralla*,* RF39*,* 0319-6G20*,* and WCHB1-41*, and *Prevotellaceae_UCG-001* were the major enriched genera (Supplementary Table S2). The PCoA plot of Bray-Curtis distance dissimilarity matrices showed no clustering of prokaryotic community structure based on doses (Fig. 3c) which was supported by PERMANOVA test with no significant difference (*p* > 0.05). The relative abundances of Verrucomicrobiota at the phylum level were significantly different (*p* < 0.05) between different doses (supplementary Figure S3). Compared to the control (dose 0) (Figs. 3d), DFS at different doses had a positive effect on the relative abundance of Proteobacteria and a negative effect on Bacteroidota, Firmicutes, Spirochaetota, and Patescibacteria, and an inconsistent effect on Verrucomicrobiota, and Cyanobacteria.

## Discussion

This study aimed to investigate the impact of specific compounds BES, HoC, and DFS on metabolic synthesis pathways of methane, acetate, and propionate-producing microbes in the context of anaerobic rumen fermentation. The rumen fermentation process involves three primary biochemical pathways, namely methanogenesis, acetogenesis, and propionigenesis, from which VFAs such as acetate, propionate, and butyrate, in addition to methane as a byproduct, are produced [32]. By targeting these pathways with different compounds, the rumen fermentation process was manipulated in this study, leading to alterations in the VFA, CH_4_, and prokaryote composition. To achieve this, BES was used as an additive to directly inhibit methane production by targeting methyl coenzyme M-reductase [33] and possibly heterdodisulfide reductase [34], while HoC [12] and DFS [35] were used as alternative hydrogen sinks.

The results of the study indicated that BES was successful in reducing methane production without any significant impact on dDM. However, BES notably decreased pH, and concentrations of acetic, isobutyric, valeric and total volatile fatty acids (tVFAs), while concentrations of propionic and butyric acids increased compared with the control MS. The increase in propionate concentration could be the result of an increased H_2_ concentration in the rumen fluid and the subsequent decrease in the pH as supported by Ungerfeld [36]. The thermodynamics of fermentation processes that create or consume H_2_ are affected by concentrations of dissolved H_2_ in the rumen fluid. When dissolved H_2_ concentrations are low, the synthesis of acetate and butyrate is thermodynamically favored, whereas propionate production is thermodynamically favorable when dissolved concentrations of H_2_ are high [7].

Similar to our research, earlier reports [36–38]found that BES decreased CH_4_ production by 95% during 24 to 72 h of fermentation with differing rumen fluid sources, differing substrates and differing dose concentrations. However, contrary to our research, Lee et al. [38] found that BES decreased the total gas production, but tVFA was unaffected. Similar to our study [16, 38] found that the propionate concentration was increased, and the bacterial population was not affected by BES treatment, whereas the methanogen population was decreased. Galindo et al. [39] found out BES reduced the abundance of cellulolytic bacteria, while our study exhibited inconsistent results. We observed that BES had a positive effect on Bacteroiodota, a negative effect on Firmicutes and a mixed effect on Proteobacteria. In ruminants, Bacteroidetes and Firmicutes are predominant phyla. However, it is possible that the in-vitro conditions are more favorable for Proteobacteria compared to other phyla as the genus *Ruminobacter* is enriched in presence of BES.

Methyl-CoM reductase (*Mcr*) is the enzyme that mediates the last step of methanogenesis. CoM (2-mercaptoethanesulfonic acid) is a crucial cofactor that serves as the methyl group carrier [40]. Among several halogenated and sulfonated compounds [41], BES is a structural analog of CoM, and it can specifically inhibit *Mcr* activity and significantly decrease methane production [42]. Nevertheless, the use of halogenated compounds such as BES must be approached with caution, as some methanogens have been shown to develop resistance to BES [43, 44]. Additionally, it is important to keep in mind that this compound is not approved for use as a feed additive for live animals and further research in vivo is needed.

The rumen ecosystem comprises methanogen and nonmethanogenic microorganisms that compete for H_2_. While nonmethanogenic microorganisms utilize various electron acceptors, including carbon dioxide (CO_2_), sulfate, nitrate, and fumarate, they are less efficient at removing H_2_ from the rumen environment than methanogens [10, 13, 45]. HoC and DFS were used as electron acceptors to decrease the abundance of methanogens by shifting H_2_ toward reductive acetogenesis and succinate pathways, and both chemicals were able to affect the fermentation parameters in this study. In this experiment ethanol was used to improve the compounds solubility, but it is important to use it minimally as it can impact rumen fermentation and microorganisms. Similar to our research, Jin et al. [46] found that HoC when used in a high forage diet, reduced CH_4_, dDM, TGP, and tVFA. Our research shows a ~ 9% decrease in dDM and ~ 5% decrease in tVFA when the control was compared with the highest dose of HoC. This outcome resulted from the H_2_ shift toward acetic acid production, as evidenced by the elevated concentrations of acetic acid compared to the control group. Reductive acetogenesis is carried out by homoacetogens, which can reduce CO_2_ using H_2_ to produce acetate by the Wood-Ljungdahl pathway [10, 47]. While their abundance is lower than methanogens, providing the electron donor to support the Wood-Ljungdahl pathway may increase homoacetogen activity and reduce methane production [48]. However, our study did not find any linear or quadratic correlation between HoC dose and acetic acid concentration, indicating no significant impact of HoC treatment. While our findings indicate an increase in acetate percentage, we cannot definitively conclude that p-hydrocinnamic acid (HoC) targets explicitly the reductive acetogenesis pathway. This argument is based on the study by Cord-Ruwisch et al. (1988) [12], but without employing techniques such as gene expression analysis, enzyme activity assays, or stable isotope tracing, these findings remain limited.

No significant difference was observed in dDM between the control and various doses of DFS, indicating that DFS did not have any adverse effects on the microbial hydrolysis of plant structural carbohydrates during 48 h of rumen microbial fermentation. The addition of DFS increased the pH, propionate, and total volatile fatty acids (tVFAs) concentration, while it reduced methane production, suggesting that the fermentation process may have shifted toward a hydrogen sink through the succinate pathway. It is important to note that fumarate is required for the synthesis of succinate, therefore, the addition of fumarate can function as an external electron acceptor, resulting in increased succinate formation and propionate production [2]. Propionate production in the rumen provides alternate sinks for H_2_ disposal and is stoichiometrically related to decreased methanogenesis [49], which is consistent with the findings of this research. A similar study by Liu et al. [50] demonstrated that combining 3-NOP with fumarate reduced methane production and synergistically increased propionate concentration, alongside a decrease in the archaeal population. In contrast to this finding, our research showed that the relative abundance of archaea decreased only with a dose of 10 mmol/L DFS. Newbold et al. [13] found that 6.5 mmol/L sodium fumarate captured 44% of the H_2_ used for CH_4_ formation during grass hay and concentrate (50:50) rumen fermentation in a RUSITEC continuous rumen fermentation system. In addition, Li et al. [35] found that CH_4_ production linearly decreased as the dose of propionate precursor (malate and fumarate) increased from 0 to 24 mM. However, the dDM was reduced as the fumarate dose increased, which is contrary to the findings of this research.

This study did not observe any significant impact on alpha and beta diversity of prokaryotic communities from the H_2_ sink compounds (HoC and DFS) and their doses. The core prokaryote community was composed of Firmicutes, Bacteroidetes, and Proteobacteria, which is consistent with the findings of Henderson et al. [51]. This core prokaryote community remained unchanged regardless of the treatment and dosage administered. However, it was observed that the H_2_ sink compounds had an impact on the relative abundance of certain bacterial phyla, such as Firmicutes, Bacteroidetes, and Proteobacteria, when compared to the control. Specifically, HoC was found to have a positive effect on Proteobacteria, and Firmicutes, while DFS had a positive effect on Proteobacteria compared to the control. According to a study by Kersters et al. [52], Proteobacteria play a role in the production of acetate and propionate. The current study revealed that there was a decrease in dDM with the increasing dose of HoC and an increase in propionate concentrations in DFS with no change in dDM. In addition, HoC increased Proteobacteria and acetic acid concentration. This may be due to the presence of HoC as an electron acceptor, resulting in the fixation of CO_2_ and H_2_ through the Wood-Ljungdahl pathway, as well as the presence of DFS as an electron acceptor, leading to the fixation of H_2_ in the succinate/randomizing pathways.

Hydrogen, a byproduct of rumen fermentation, plays a crucial role in regulating the rumen fermentation process [36]. The accumulation of dissolved H_2_ in the rumen can hinder fermentation and microbial metabolism [48]. Methanogenesis is the most efficient process for H_2_ disposal, but inhibiting it results in the need for an alternative H_2_ sink [53]. Several commercial products are available to inhibit methanogens [40, 54, 55], with more expected to come. Nevertheless, to balance the rumen ecosystem, additional alternative H_2_ sinks are required to take over the role of methanogens. One potential approach tested and shown in the current study is to increase the activity of homoacetogens, which are already present in the rumen, by providing electron acceptors for reductive acetogenesis. Furthermore, enhancing propionigenesis to divert H_2_ from CH_4_ to propionate is a promising mitigation strategy. It is imperative to explore these alternatives to ensure that the rumen ecosystem is in balance and that sustainable production is maintained.

In this study, we used 16S rRNA amplicon sequencing to study the rumen microbiome taxonomy. However, 16S rRNA amplicon sequencing has limitations, such as low resolution for closely related species, primer biases, abundance quantification inaccuracies, and artifacts like chimeras. In addition, the additives that have been studied in this research were tested in conjunction with simple basal diets (maize silage), which might not reflect in vivo conditions.

## Conclusions

This study confirms that BES can effectively inhibit methanogenesis without compromising dry matter degradability. Additionally, the study found that HoC and DFS can redirect H_2_ toward the synthesis of acetate and propionate. Importantly, none of the compounds altered the core prokaryote composition and structure. Based on the current study, the recommended doses, to reduce methane during in-vitro rumen fermentation for BES, HoC, and DFS were determined to be 2.5 mmol/L, 5 mmol/L, and 10 mmol/L, respectively. However, further research is needed to fully understand the interactive effects of methane inhibition compounds, such as BES, in conjunction with H_2_ sink compounds like HoC and DFS. Caution is advised when using halogenated compounds like BES, as some methanogens have developed resistance. Additionally, BES is not approved for use as a feed additive for live animals, requiring further in vivo research.

## Electronic supplementary material

Below is the link to the electronic supplementary material.

Supplementary Material 1

## Data Availability

The microbiome datasets analysed in this study were submitted to the NCBI Sequence Read Archive (SRA) under the accession number PRJNA1028548.

## Abbreviations

ASV: Amplicon sequence variants
BES: Sodium 2-bromoethanesulfonate
CP: Crude protein
dDM: Degraded dry matter
DFS: Sodium fumarate dibasic
DM: Dry matter
FAO: United nation Food and Agriculture Organization
GC: Gas chromatography
HoC: p-hydrocinnamic acid
MS: Maize silage
STP: Standard temperature and pressure
VFAs: Volatile Fatty Acids
