# Supplementary material for: Investigating dose-dependent effects of chemical compounds targeting rumen fermentation pathways using an in-vitro rumen fermentation system
Source: BMC Microbiol. 2025 May 27;25:330. doi: 10.1186/s12866-025-03969-7 (PMC12107880; doi:10.1186/s12866-025-03969-7)
Supplement: Supplementary file 1 — Supplementary Material 1 [file 12866_2025_3969_MOESM1_ESM.docx]

**Supplementary Table**

Table S1. Experiment information

| **Treatment** | **Dose (mmol/L)** | **No of fermentation (batch)** | **Bottles in each Treatments** | **Remarks** |
| --- | --- | --- | --- | --- |
| BES | 0 | 3 | 3 |  |
|  | 2.5 | 3 | 3 |  |
|  | 5 | 3 | 3 |  |
|  | 10 | 3 | 3 |  |
| HoC | 0 | 3 | 3 | 0.5 ml (80% ethanol) |
|  | 5 | 3 | 3 | 0.5 ml (80% ethanol) |
|  | 10 | 3 | 3 | 0.5 ml (80% ethanol) |
| DFS | 0 | 3 | 3 | 0.5 ml (80% ethanol) |
|  | 5 | 3 | 3 | 0.5 ml (80% ethanol) |
|  | 10 | 3 | 3 | 0.5 ml (80% ethanol) |
|  | 20 | 3 | 3 | 0.5 ml (80% ethanol) |

BES: Sodium 2-bromoethanesulfonate, HoC: p-hydrocinnamic acid, DFS: Sodium fumarate dibasic.

**Supplementary figures:**

**
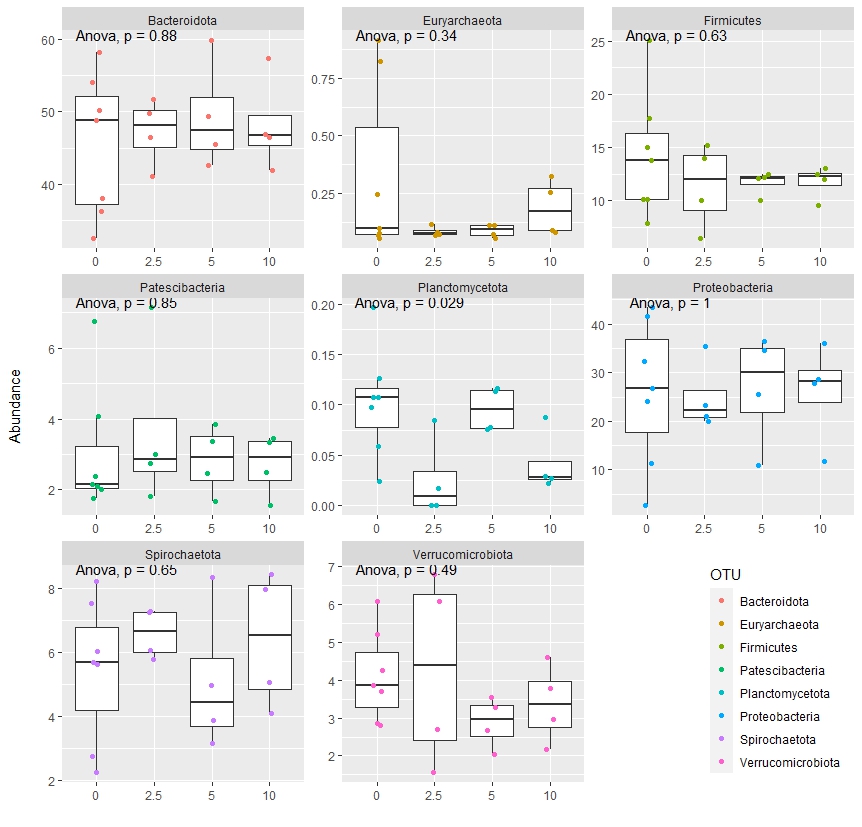
**

**Figure S1.** BES individual relative abundance.


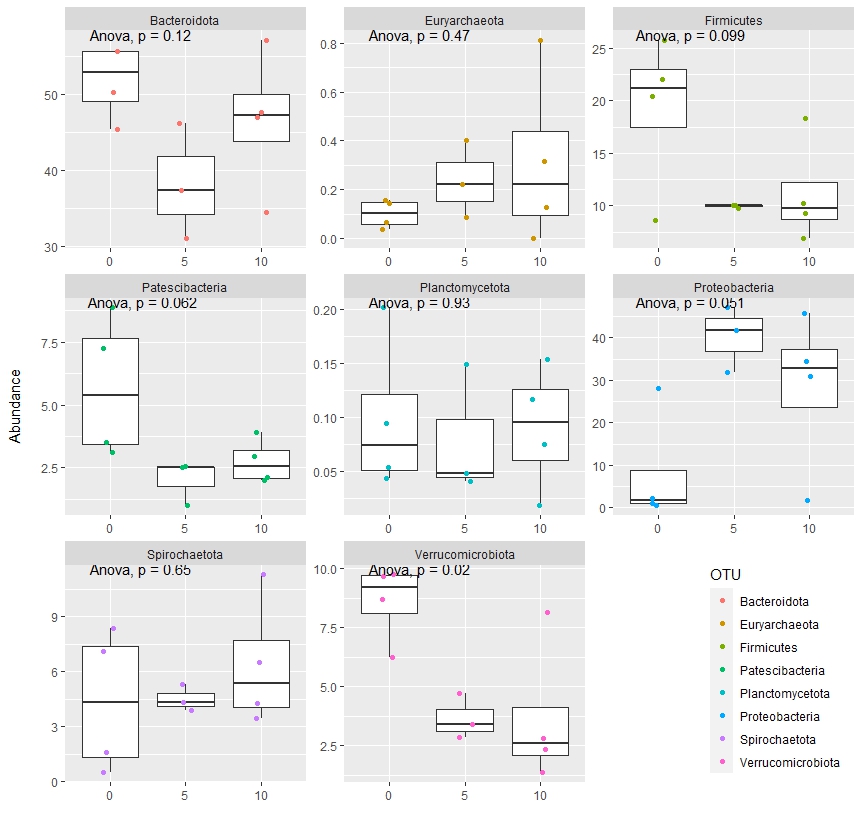


**Figure S2.** HoC individual relative abundance.


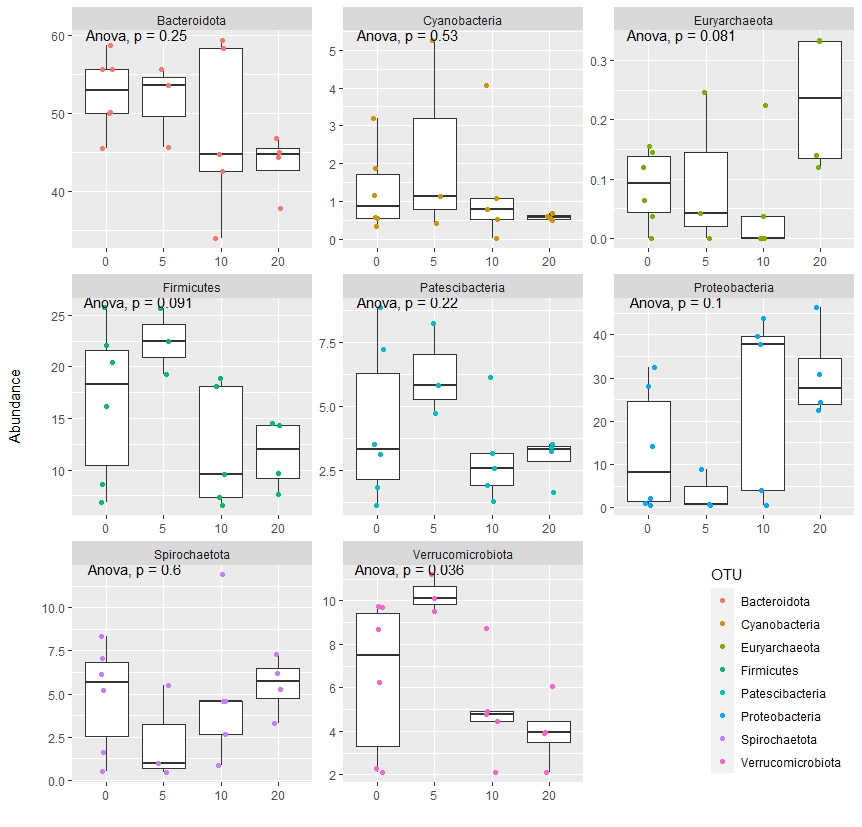


**Figure S3.** DFS individual relative abundance.

**
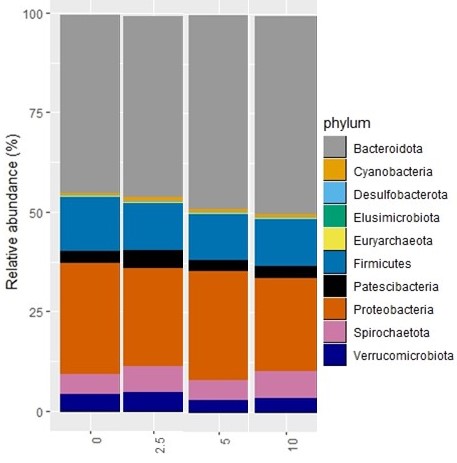
**

**Figure S4:** Prokaryote community composition at genus level of BES.


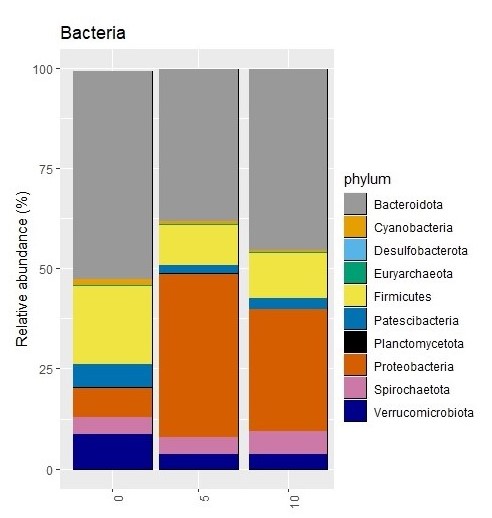


**Figure S5:** Prokaryote community composition at genus level of HoC.

**
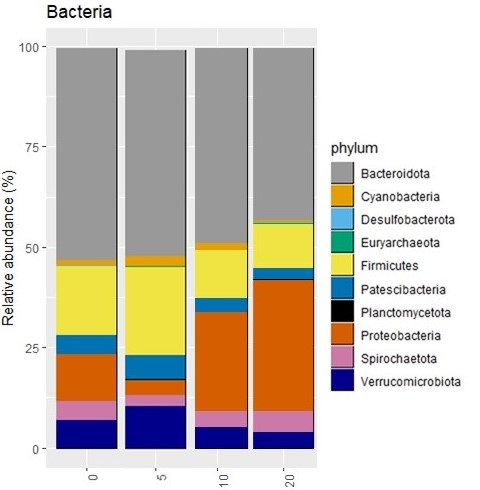
**

**Figure S6:** Prokaryote community composition at genus level of DFS.


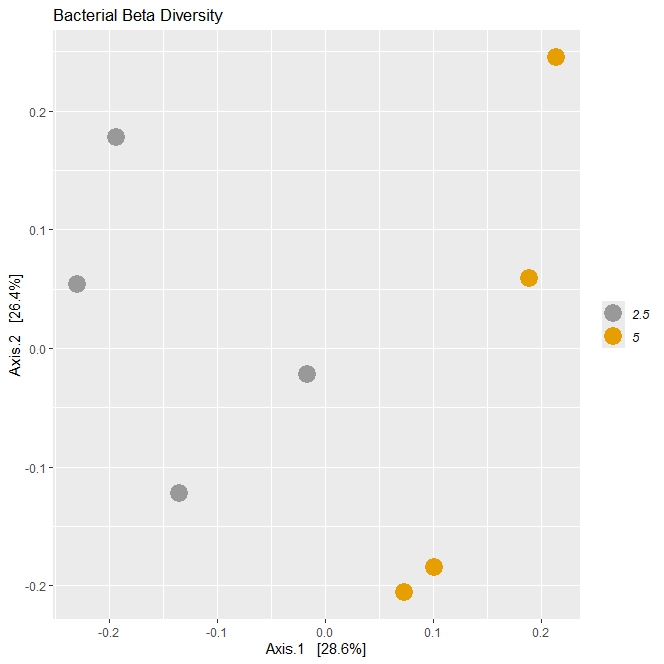


**Figure S7.** Community beta diversity of 2-bromoethanesulfonate (BES) between 2.5 and 5 doses.

**
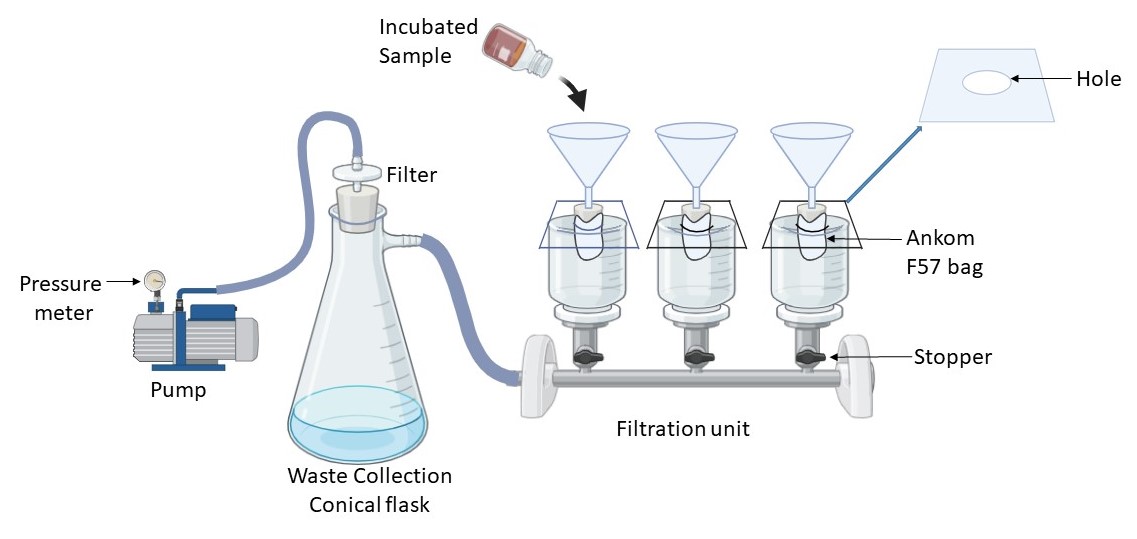
**

**Figure S8.** Schematic representation of filtration system used in this experiment.

**Table S2.** Enrich prokaryote (p <0.001) in sodium 2-bromoethanesulfonate (BES), sodium fumarate dibasic (DFS) and p-hydrocinnamic acid (HoC) treatment.

| **Compound** | **log2FoldChange** | **lfcSE** | **Phylum** | **Genus** | **Contrast** |
| --- | --- | --- | --- | --- | --- |
| BES | 19.93 | 3.80 | Firmicutes | <NA> | 5 vs 10 |
|  | 21.96 | 3.01 | Firmicutes | probable_genus_10 | 5 vs 10 |
|  | 20.13 | 4.26 | Bacteroidota | Rikenellaceae_RC9_gut_group | 5 vs 10 |
|  | 22.45 | 4.01 | Bacteroidota | Rikenellaceae_RC9_gut_group | 5 vs_2.5 |
|  | 21.32 | 4.07 | Proteobacteria | Ruminobacter | 5 vs 10 |
|  | 21.22 | 4.56 | Bacteroidota | Bacteroidales_UCG-001 | 5 vs 10 |
|  | 22.61 | 4.54 | Bacteroidota | Bacteroidales_UCG-001 | 5 vs_2.5 |
|  | 21.13 | 4.07 | Bacteroidota | SP3-e08 | 0 vs 5 |
|  | 20.04 | 4.07 | Bacteroidota | F082 | 0 vs 5 |
|  | 20.83 | 3.88 | Spirochaetota | Treponema | 0 vs 5 |
|  | -32.20 | 4.41 | Spirochaetota | Treponema | 5 vs 10 |
|  | 21.88 | 3.92 | Firmicutes | UCG-002 | 5 vs 2.5 |
|  | 23.37 | 4.33 | Verrucomicrobiota | WCHB1-41 | 5 vs 2.5 |
| DFS | 22.92 | 4.78 | Bacteroidota | F082 | 5 vs 10 |
|  | 22.83 | 4.48 | Bacteroidota | F082 | 0 vs 5 |
|  | 23.14 | 4.49 | Proteobacteria | Succinivibrionaceae_UCG-002 | 0 vs 5 |
|  | 23.23 | 4.56 | Bacteroidota | Prevotella | 5 vs 10 |
|  | 22.06 | 4.48 | Bacteroidota | Prevotella | 0 vs 5 |
|  | 22.51 | 4.70 | Firmicutes | Butyrivibrio | 5 vs 20 |
|  | 21.77 | 4.08 | Proteobacteria | Sutteralla | 0 vs 5 |
|  | 21.52 | 4.29 | Bdellovibrionota | 0319-6G20 | 5 vs 20 |
|  | 22.10 | 4.29 | Firmicutes | RF39 | 5 vs 10 |
|  | -19.33 | 4.09 | Bacteroidota | F082 |  |
|  | 21.20 | 4.04 | Verrucomicrobiota | WCHB1-41 | 0 vs 5 |
|  | -20.89 | 4.29 | Bacteroidota | Prevotellaceae_UCG-001 | 0 vs 5 |
| HoC | -23.61 | 3.84 | Bacteroidota | F082 | 0 vs 5 |
|  | -20.65 | 3.79 | Bacteroidota | Prevotella | 0 vs 5 |
|  | 21.16 | 3.79 | Bacteroidota | Prevotellaceae_UCG-001 | 0 vs 5 |
|  | -21.34 | 3.79 | Bacteroidota | Prevotellaceae_UCG-001 | 0 vs 5 |
|  | 21.92 | 3.84 | Firmicutes | UCG-002 | 0 vs 5 |
|  | -21.03 | 3.52 | Firmicutes | Ruminococcus | 0 vs 5 |
